# Translation, cross-cultural adaptation, and semantic validation of the Spine Oncology Study Group Outcomes Questionnaire 2.0 (SOSGOQ 2.0) into Brazilian Portuguese: a qualitative validation study

**DOI:** 10.31744/einstein_journal/2026AO2116

**Published:** 2026-05-27

**Authors:** Guilherme Pianowski Pajanoti, Bruno Braga Roberto, Fernando Padilla-Lichtenberger, Federico Landriel, Santiago Hem, Nelson Astur

**Affiliations:** 1 Hospital Israelita Albert Einstein São Paulo SP Brazil Hospital Israelita Albert Einstein, São Paulo, SP, Brazil.; 2 Hospital Israelita Albert Einstein Hospital Municipal Gilson de Cássia Marques de Carvalho São Paulo SP Brazil Hospital Municipal Gilson de Cássia Marques de Carvalho; Hospital Israelita Albert Einstein, São Paulo, SP, Brazil.; 3 Hospital Italiano de Buenos Aires Buenos Aires Argentina Hospital Italiano de Buenos Aires, Buenos Aires, Argentina.

**Keywords:** Spinal neoplasms, Metastases neoplasms, Quality of life, Semantics, Validation studies, Surveys and questionnaires

## Abstract

**Objective:**

To translate, culturally adapt, and semantically validate the Brazilian Portuguese version of the Spine Oncology Study Group Outcomes Questionnaire 2.0 (SOSGOQ 2.0) through cognitive interviews with patients with spinal metastases.

**Methods:**

Following the guidelines of Beaton et al., the translation process included forward translation by two independent Brazilian translators, back translation by two native English speakers, expert committee review, and reconciliation. The final version underwent semantic validation through cognitive interviews with five patients with spinal metastases at a Brazilian public cancer center. Following Consolidated Criteria for Reporting Qualitative Research guidelines, semi-structured interviews assessed comprehensibility, relevance, and cultural appropriateness. All interviews were recorded, transcribed, and analyzed using thematic content analysis.

**Results:**

All participants (mean age 58±12 years, 60% male) completed the questionnaire successfully. Thematic analysis identified three main themes. High comprehensibility was observed, as all items were clearly understood without ambiguity. Cultural relevance emerged because the questions reflected the Brazilian healthcare context appropriately. Clinical applicability was also evident, as participants recognized the relevance of the symptoms addressed in the questionnaire to their condition. The mean completion time was 12±3 min, and no linguistic modifications were required. Participant feedback demonstrated semantic equivalence and acceptability of the translated instrument.

**Conclusion:**

The Brazilian Portuguese SOSGOQ 2.0 demonstrated strong semantic validity and cultural appropriateness in this qualitative validation study. The instrument is ready for large-scale psychometric validation studies in Brazilian oncology settings.

## In Brief

This study translated and semantically validated SOSGOQ 2.0 into Brazilian Portuguese through cognitive interviews with patients diagnosed with spinal metastases. The instrument demonstrated high clarity, cultural appropriateness, and full completion, supporting clinical use and future large-scale psychometric validation.

## Highlights

■ Brazilian Portuguese translation of SOSGOQ 2.0 scale.
■ First semantic clinical validation of Brazilian SOSGOQ 2.0.
■ Cognitive interviews confirmed clarity and cultural appropriateness.
■ 100% completion with no need for clarification.
■ Ready for large-scale psychometric validation.

## Graphical abstract

**Figure:**
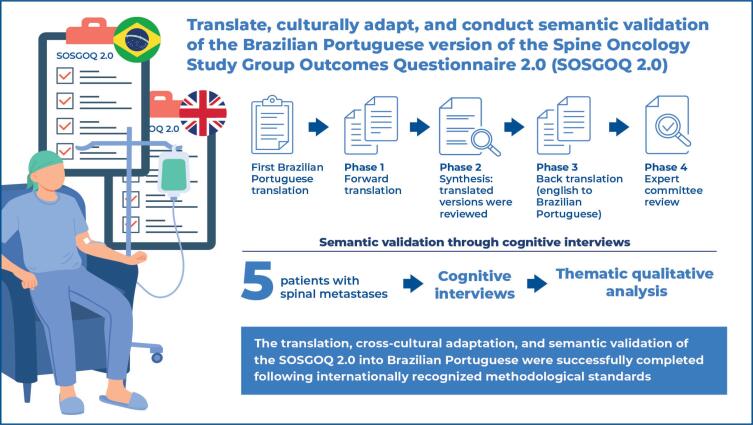

## INTRODUCTION

Cancer is among the leading causes of mortality worldwide.^(1)^ The incidence of symptomatic spinal metastases has increased in recent years, largely as a result of advances in cancer treatment and prolonged patient survival. Expected patient survival is a key determinant of therapeutic decision-making.^(1)^ In Brazil, secondary neoplasms affecting the spine have been among the most prevalent forms of bone metastasis since the early 2000s.^(2)^ Metastatic spinal disease represents a major public health concern, particularly within the public healthcare system.^(1–3)^ It is the most common type of spinal tumor treated at major Brazilian institutions and is associated with substantial morbidity, mortality, and healthcare resource utilization.^(2,3)^ Management of these patients frequently requires a multidisciplinary approach that includes surgery, radiotherapy, and chemotherapy. Surgical intervention is often necessary to restore or preserve neurological function, stabilize the spine, and alleviate pain.^(1–4)^

The assessment of health-related quality of life has become a fundamental component of contemporary oncological and palliative care, particularly in conditions that substantially impair physical function, such as metastatic spinal lesions.^(5)^ Nevertheless, measuring health-related quality of life-related clinical outcomes remains challenging, largely because of the lack of instruments capable of specifically capturing the limitations experienced by this patient population.

Although generic instruments such as the SF-36 and EQ-5D, as well as disease-specific indices such as the Oswestry Disability Index and Neck Disability Index, are widely used, none were originally developed for patients with spinal tumors. These tools provide a general overview of health status, but they often fail to capture the nuanced symptomatology and functional impairments associated with metastatic spinal disease.^(6)^ To address this gap, the Spine Oncology Study Group developed the Spine Oncology Study Group Outcomes Questionnaire (SOSGOQ). The revised version, SOSGOQ 2.0, improves upon the original instrument by separating items related to autonomic function and reorganizing domains to better reflect the lived experiences of patients.^(7,8)^ International validation studies have demonstrated that SOSGOQ 2.0 exhibits superior psychometric properties compared with generic measures and is more sensitive in detecting clinically meaningful changes.^(7,8)^ Cross-cultural translations and validations have been successfully conducted in countries such as Thailand,^(9)^ Italy,^(10)^ Germany,^(11)^ and China,^(12)^ further supporting its global relevance and applicability.

In 2024, Batista et al. published the first Brazilian Portuguese translation of SOSGOQ 2.0.^(13)^ Although this work provided an important foundation through translation and cross-cultural adaptation using the Reichenheim et al. framework,^(14)^ the authors acknowledged as a limitation that "the instrument was not submitted to psychometric validation" and recommended future clinical validation studies. This represents a critical gap, because semantic validation through clinical application is essential before large-scale psychometric validation can be performed.

Accordingly, the present study aimed to advance the Brazilian validation of SOSGOQ 2.0 by conducting the first clinical application and semantic validation of the instrument through cognitive interviews with patients diagnosed with spinal metastases. This study employed the internationally recognized Beaton et al. methodology^(15)^ and followed the Consolidated Criteria for Reporting Qualitative Research (COREQ) guidelines for qualitative research^(16)^ to ensure rigorous semantic validation and establish a foundation for future psychometric studies.

## OBJECTIVE

This study aimed to translate, culturally adapt, and clinically apply SOSGOQ 2.0 for use in Brazilian Portuguese. Specifically, the study evaluated the psychometric properties of the translated version, including internal consistency and construct validity, in patients with spinal tumors to ensure that the instrument is reliable, reproducible, and culturally appropriate for clinical and research applications in Brazil.

## METHODS

### Study design and setting

This methodological, prospective, single-center study focused on translation and semantic validation of the Brazilian Portuguese SOSGOQ 2.0 through cognitive interviews with patients diagnosed with spinal metastases. The study was approved by the Research Ethics Committee of *Hospital Israelita Albert Einstein* (CAAE: 83235924.2.0000.0071; #7.364.227) and conducted by the AO Spine Latin America Tumor Study Group (Grant ID 295). The study followed the COREQ guidelines.^(16)^

### Research team and reflexivity

The principal investigator, a bilingual orthopedic spine surgeon, conducted all cognitive interviews. No prior therapeutic relationship existed between the interviewer and the participants, which helped minimize response bias. Training in qualitative interview techniques was completed before data collection.

The interviewer disclosed the researcher role rather than a treating physician role and explained the academic objectives of the study to promote participant comfort in providing honest feedback about the questionnaire.

### Translation and cross-cultural adaptation process

The study followed the methodology proposed by Beaton et al.^(15)^ for cross-cultural adaptation of instruments and was structured into sequential phases.

In Phase 1 (forward translation), two independent Brazilian translators fluent in English and blinded to the study objectives performed conceptual translations of the original SOSGOQ 2.0, prioritizing equivalence of meaning rather than literal word-for-word translation.

In Phase 2 (synthesis), the translated versions were jointly reviewed by the two translators and two spine surgeon researchers until consensus was reached on the first Brazilian Portuguese version.

In Phase 3 (back translation), two native English-speaking translators fluent in Portuguese and blinded to the original instrument independently translated the Portuguese version back into English. These versions were compared and reconciled by consensus to produce a single back-translated version.

In Phase 4 (expert committee review), the English back-translated version underwent review by the original Brazilian translators after a 30-day interval to ensure consistency with the original instrument. This process resulted in consolidation of the final Portuguese version of SOSGOQ 2.0 (Appendix 1).

### Semantic validation through cognitive interviews

The final Portuguese version of SOSGOQ 2.0 was administered to five patients with spinal metastases treated at Hospital Vila Santa Catarina, a public oncology hospital managed by *Hospital Israelita Albert Einstein*.

Inclusion criteria included age >18 years, literacy in Portuguese, diagnosis of spinal metastases treated with surgery and/or radiotherapy, and the ability to provide informed consent.

Exclusion criteria included cognitive impairment that prevented provision of informed consent, inability to read Portuguese, and acute medical instability.

Purposive sampling was used to recruit patients with diverse demographic characteristics and disease presentations to ensure broad representation of the target population.

All interviews were conducted in a private consultation room at *Hospital Municipal Gilson de Cássia Marques de Carvalho*, separate from clinical treatment areas, to ensure patient comfort and confidentiality.

### Cognitive interview methodology

Cognitive interviews followed an established methodology based on the frameworks proposed by Willis^(17)^ and Beatty et al.^(18)^ for questionnaire validation, using think-aloud and verbal probing techniques.

After independent completion of the printed questionnaire, each participant underwent a semi-structured cognitive interview lasting 10-15 min. The interview aimed to assess clarity and comprehensibility of each item, evaluate appropriateness of terminology, and identify any doubts, ambiguities, or discomfort experienced during completion.

A standardized interview guide covered five domains: general comprehension, cultural appropriateness, clinical relevance, adequacy of response options, and overall assessment (Appendix 2).

All interviews were conducted individually. The principal investigator documented responses in detailed field notes, and questionnaire completion time was recorded to assess feasibility.

### Data analysis

Interview data underwent thematic content analysis according to established qualitative research methodology.^(19)^ Systematic coding identified recurring themes related to comprehensibility, cultural relevance, and clinical applicability.

Feasibility assessment included quantitative measures such as questionnaire completion time, completion rate, and the frequency of clarification requests.

### Ethical considerations

All participants received information about the objectives, procedures, risks, and benefits of the study and provided written informed consent before participation. The study protocol was reviewed and approved by the institutional research ethics committee.

## RESULTS

### Participant characteristics

Five patients completed the study between March and April 2024. All eligible patients agreed to participate, resulting in a participation rate of 100%. Participant demographics and clinical characteristics appear in table 1.

**Table 1:** Participant demographics and clinical characteristics.

Characteristic		n=5
Age, mean±standard deviation (range)		58±12 (42-74)
Sex, n (%)		
	Male	3 (60)
	Female	2 (40)
Education, n (%)		
	Primary school	2 (40)
	High school	2 (40)
	University	1 (20)
Primary tumor location, n (%)		
	Breast	2 (40)
	Lung	1 (20)
	Prostate	1 (20)
	Renal	1 (20)
Spinal level affected, n (%)		
	Cervical	1 (20)
	Thoracic	2 (40)
	Lumbar	2 (40)
Treatment received, n (%)		
	Surgery + radiotherapy	3 (60)
	Radiotherapy alone	2 (40)

### Questionnaire completion feasibility

All five participants (100%) completed the 20-item SOSGOQ 2.0 without assistance or clarification during the completion process. No items were left blank, and no confusion requiring intervention occurred.

The mean completion time was 12±3 min (range: 9-15 min). All participants completed the questionnaire within the anticipated timeframe for clinical feasibility.

No participant requested clarification of the instructions or item content during questionnaire completion, which indicates adequate clarity of the translated version.

### Qualitative interview analysis

Thematic analysis of the cognitive interviews identified three primary themes related to semantic validity and cultural appropriateness of the Brazilian Portuguese SOSGOQ 2.0.

High comprehensibility and linguistic clarity emerged as a consistent theme across interviews. All participants demonstrated clear understanding of the questionnaire items without the need for clarification, repetition, or explanation. The translated terminology was consistently understood across different educational levels.

Cultural and contextual relevance also emerged from participant feedback. Participants confirmed that the questionnaire items appropriately reflected healthcare experiences and cultural contexts in Brazil. The translated version maintained relevance within the Brazilian public healthcare system.

Clinical applicability and symptom recognition represented a third major theme. Participants recognized a clear correlation between the questionnaire items and the clinical symptoms and functional limitations associated with spinal metastases. All participants confirmed the clinical relevance of the assessed domains.

### Linguistic and content assessment

None of the participants suggested modifications to wording, phrasing, or terminology. The translated version maintained conceptual equivalence with the original instrument while using culturally appropriate Brazilian Portuguese expressions. Participants confirmed that all 20 items addressed symptoms and functional limitations relevant to their experience with spinal metastases, and no suggestions were made for item addition or removal.

### Modifications and adaptations

No linguistic modifications to the translated text were required based on feedback from the cognitive interviews. Semantic validation therefore confirmed adequacy of the translation process.

No cultural adaptations were considered necessary, as participants confirmed appropriateness of all content within the Brazilian healthcare context.

## DISCUSSION

### Methodological advances beyond the previous Brazilian translation

This study significantly extends the work of Batista et al.^(13)^ through several methodological enhancements. Batista et al. provided translation and cross-cultural adaptation using the Beaton et al. framework,^(15)^ whereas the present study advances the field in several ways. First, the study employed the internationally recognized Beaton et al. methodology,^(15)^ which includes rigorous forward and back translation with expert committee review. Second, the study conducted the first clinical application of the instrument in patients diagnosed with spinal metastases. Third, systematic cognitive interviews followed COREQ guidelines^(16)^ with thematic analysis of qualitative data. Fourth, the study provided empirical evidence of semantic validity through direct patient feedback.

Batista et al. also acknowledged as a limitation that "the instrument was not submitted to psychometric validation" and recommended future studies to conduct clinical validation. The present study addresses this gap by establishing an essential semantic validation foundation for future large-scale psychometric investigations. The combination of rigorous translation methodology and clinical validation through patient cognitive interviews provides stronger evidence supporting linguistic equivalence and cultural appropriateness of the Brazilian Portuguese SOSGOQ 2.0.

### Semantic validation and clinical applicability

The cognitive interview methodology used in this study provides important evidence of semantic validity and the extent to which questionnaire items are understood as intended by the target population.^(20)^ The findings indicate that the Brazilian Portuguese SOSGOQ 2.0 demonstrates high semantic validity, as all participants understood the items clearly and recognized their clinical relevance. This aspect is particularly important for patient-reported outcome measures, in which misunderstanding of items may compromise data quality and clinical utility.^(21)^

The average completion time of 12 min supports feasibility for use in busy oncology settings. This duration is comparable to that reported for other validated spine-specific questionnaires and remains acceptable for routine clinical use.^(7,8)^ The absence of requests for assistance or clarification during completion further indicates that the instrument can be reliably self-administered across different educational levels.

### Cultural appropriateness and Brazilian healthcare context

An important finding of this study was confirmation of cultural appropriateness within the Brazilian healthcare context. Participants indicated that the questionnaire items reflected their experiences within the Brazilian public healthcare system. Cultural alignment represents a key requirement for patient-reported outcome measures, because cultural mismatch may compromise validity even when linguistic translation remains accurate.^(22)^

The diversity of participant educational backgrounds, which ranged from primary school to university level, and the consistent understanding of the questionnaire items suggest broad applicability across different socioeconomic groups in Brazil. This aspect is particularly relevant for the Brazilian public healthcare system, which serves a highly diverse patient population.

### International collaboration and regional impact

This study was conducted in collaboration with an international group of researchers and members of the Latin America Spine Tumor Study Group. The inclusion of authors from different countries, including contributors whose native language is not Portuguese, reflects the original objective of developing a standardized methodological instrument suitable for multicenter studies across Latin America. This collaboration strengthened methodological rigor, expanded the cross-cultural applicability of the translated version, and promoted scientific integration across the region.

The validated Brazilian Portuguese SOSGOQ 2.0 may also prove applicable to other Portuguese-speaking countries, although this possibility requires confirmation through future verification studies. Such expansion could facilitate international research collaboration and comparative effectiveness studies across Portuguese-speaking populations.

### Study limitations and psychometric validation requirements

This study represents the semantic validation phase of instrument adaptation recommended by the Consensus-based Standards for the Selection of Health Measurement Instruments (COSMIN) guidelines for patient-reported outcome measures.^(23)^ Although the qualitative validation provides important evidence regarding comprehensibility and cultural appropriateness, comprehensive psychometric validation, including assessment of reliability, validity, and responsiveness, requires larger sample sizes according to COSMIN standards.

According to COSMIN methodology, semantic validation studies typically include 5-10 participants for cognitive interviews to evaluate comprehensibility. In contrast, psychometric validation requires substantially larger samples, with at least 100 patients for reliability assessment and more than 150 patients for construct validity testing.^(23,24)^ The present study therefore provides an important foundation for future such larger-scale quantitative validation studies, which are currently underway with expanded patient recruitment across multiple Brazilian centers.

The single-center design, although appropriate for semantic validation, limits generalizability across different regions and healthcare settings in Brazil. Future multicenter studies should confirm these findings across diverse geographical and institutional contexts.

### Clinical implications and future directions

The availability of a semantically validated Brazilian Portuguese SOSGOQ 2.0 enables spine oncology centers in Brazil to collect standardized patient-reported outcome data, which supports evidence-based clinical decision-making and research. The instrument can facilitate systematic assessment of treatment outcomes in patients with spinal metastases, comparison of therapeutic interventions within Brazilian healthcare settings, participation in international multicenter studies, and development of Brazilian clinical guidelines for spinal oncology care.

Future research should focus on large-scale psychometric validation following COSMIN guidelines, establishment of Brazilian population norms and minimal clinically important differences, responsiveness testing in longitudinal studies, validation within specific cancer subtypes and treatment modalities, and implementation research in routine clinical practice settings.

The semantic validation approach used in this study follows established international precedents for questionnaire adaptation, in which qualitative assessment precedes quantitative psychometric testing.^(25)^ This sequential validation strategy ensures that linguistic and cultural barriers are addressed before large-scale statistical validation studies are undertaken.

## CONCLUSION

Translation, cross-cultural adaptation, and semantic validation of SOSGOQ 2.0 into Brazilian Portuguese were successfully completed following internationally recognized methodological standards. Cognitive interviews with patients diagnosed with spinal metastases demonstrated high semantic validity, cultural appropriateness, and clinical applicability of the Brazilian Portuguese version. All participants understood the questionnaire items, recognized their clinical relevance, and confirmed their cultural appropriateness within the Brazilian healthcare context.

These findings establish the semantic foundation required for future large-scale psychometric validation studies and support potential use of the instrument in Brazilian clinical practice and research. The validated Brazilian Portuguese SOSGOQ 2.0 represents an important advancement in spine oncology research in Brazil by enabling standardized assessment of patient-reported outcomes and facilitating participation in international research collaborations.

## Data Availability

All underlying data are contained within the manuscript.

## Appendix 2 Interview guide

**Table:**

This semi-structured interview guide was used for cognitive interviews to evaluate the Brazilian Portuguese version of SOSGOQ 2.0. The guide addressed five key domains.
1. General comprehension Were any questions difficult to understand? Did any words or phrases appear unclear or confusing? Did you need help interpreting any part of the questionnaire?
2. Cultural appropriateness Do the questions reflect experiences common in your daily life and healthcare context? Is the wording or content consistent with your culture and background? Did any items appear inappropriate or irrelevant for patients in Brazil?
3. Clinical relevance Did the questions relate to your symptoms and health condition? Were important aspects of your health problems addressed? Was anything missing that you consider relevant for patients with spinal metastases?
4. Adequacy of response options Were the answer options sufficient to express your experience? Were the response alternatives easy to understand? Did you have difficulty choosing a response for any item?
5. Overall assessment How was your overall experience completing the questionnaire? Do you have suggestions to improve clarity or relevance of the questionnaire? Would you recommend any changes for use with other patients?
